# GATA elements control repression of cardiac troponin I promoter activity in skeletal muscle cells

**DOI:** 10.1186/1471-2199-8-78

**Published:** 2007-09-17

**Authors:** Raffaella Di Lisi, Anne Picard, Simonetta Ausoni, Stefano Schiaffino

**Affiliations:** 1 Department of Biomedical Sciences, University of Padova, Padova, Italy; 2 CNR Institute of Neurosciences, University of Padova, Padova, Italy; 3 Venetian Institute of Molecular Medicine, Padova, Italy

## Abstract

**Background:**

We reported previously that the cardiac troponin I (cTnI) promoter drives cardiac-specific expression of reporter genes in cardiac muscle cells and in transgenic mice, and that disruption of GATA elements inactivates the cTnI promoter in cultured cardiomyocytes. We have now examined the role of cTnI promoter GATA elements in skeletal muscle cells.

**Results:**

Mutation or deletion of GATA elements induces a strong transcriptional activation of the cTnI promoter in regenerating skeletal muscle and in cultured skeletal muscle cells. Electrophoretic mobility shift assays show that proteins present in nuclear extracts of C2C12 muscle cells bind the GATA motifs present in the cTnI promoter. However, GATA protein complex formation is neither reduced nor supershifted by antibodies specific for GATA-2, -3 and -4, the only GATA transcripts present in muscle cells.

**Conclusion:**

These findings indicate that the cTnI gene promoter is repressed in skeletal muscle cells by GATA-like factors and open the way to further studies aimed at identifying these factors.

## Background

Establishment and maintenance of cell specification involves both activating and repressive mechanisms. While positive gene regulation by tissue-specific transcription factors is well established, the role of negatively acting transcription factors has been comparatively less investigated. For example, cardiac and skeletal muscle cells are known to co-express a large number of muscle genes, but the regulatory regions in the gene promoters differ between the two cell types. Both common regulatory factors, such as MEF2, and cardiac- or skeletal muscle-specific transcription factors, such as GATA-4 and MyoD, respectively, have been implicated as tissue-specific activators of muscle gene expression. A few studies have described the repression of muscle gene as a mechanism that control cardiac muscle restricted gene expression. Mutations in the HF-3 element (TAACCTTGAAGGC) of the ventricular myosin light chain 2 promoter leads to marked up-regulation of promoter activity in skeletal muscles of transgenic mice [1]. The transcription factor Nishéd, which binds to a GAAG/CTTC sequence, appears to account for the repression of the chicken cardiac myosin light chain 2 in skeletal muscle [2].

The cardiac troponin I (cTnI) gene encodes for the cardiac specific inhibitory subunit of the troponin complex, one of the few sarcomeric proteins that is expressed in cardiac but not skeletal muscle [3]. We have reported previously that this specificity is controlled by the proximal 5'-flanking region of the promoter (-230/+16), which drives cardiac-specific expression both in cultured cardiac cells, in terminally differentiated cardiac muscle cell in vivo and in transgenic mice [4]. Different types of transcription factors control the expression of the cTnI promoter in cardiac muscle cells. Among these, three GATA elements, that are targets of GATA-4 transcription factor, are required for promoter activity, as disruption of these elements inactivates the cTnI promoter in cultured cardiomyocytes [4]. Here we report that, surprisingly, the activity of the cTnI promoter is markedly increased in skeletal muscle by mutation or deletion of GATA sites. Nuclear extracts of muscle cells contain proteins distinct from canonical GATA factors that form specific complexes with the GATA elements present in the cTnI promoter. These findings point to a novel mechanism of repression of cardiac gene promoters in skeletal muscle involving GATA sites.

## Results

### cTnI promoter activity is increased by disruption of GATA elements in skeletal muscle cells

The proximal promoter of the cTnI gene (-230/+16) is sufficient to drive cardiac-specific expression of reporter genes both in cultured cardiomyocytes and in transgenic mice [4]. Promoter analysis showed that three types of regulatory elements are required for optimal gene activation. These include three GA-rich elements binding Sp1 and located betweeen -175 and -133, an A/T-rich motif binding MEF2 and Oct1 and lying at position -36, and three GATA elements, two of which are closely associated between -67 and -57 of the promoter whereas the third is located just upstream of the transcriptional start site. Deletion and mutation analyses showed that mutation of GATA elements inactivated the cTnI promoter in cultured cardiomyocytes [4].

**Figure 1:**
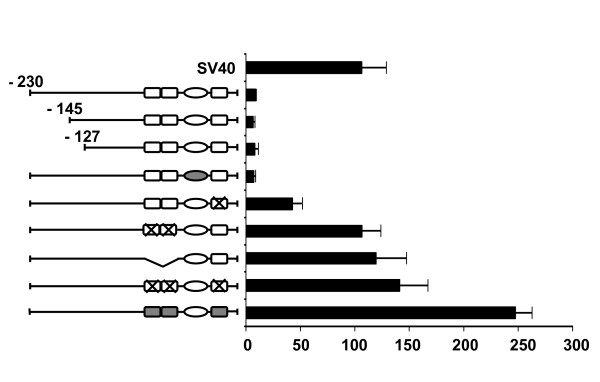
**The cTnI promoter is activated by mutation or deletion of GATA sites in skeletal muscle *in vivo***. Transfection experiments of cTnI-CAT constructs in regenerating rat soleus muscles. The -230/+16 cTnI promoter and different mutants are schematically represented on the left, the three GATA site being indicated with squares and the A/T rich element with an oval. CAT activities in transfected muscles are shown on the right. The following mutants were examined (top to bottom): -145 and -127 deletion mutants, leading to the deletion of two of three or all three GA-rich sequences, respectively; mutation of the A/T-rich sequence (filled oval); GATA → CACA mutation of the proximal GATA site (crossed square); GATA → CACA mutation of the two distal GATA sites (two crossed squares); GATA → CACA mutation of all three GATA sites (three crossed squares); deletion of the two distal GATA sites; GATA → GCCA mutation of all three GATA sites (filled squares). Note that deletion/mutation of GATA sites leads to increased activity of the cTnI promoter, up to levels even higher than the SV40 promoter. Values (means ± S.E.) are expressed as fold increase in CAT activity over the promoterless construct. Each construct was tested in 4 to 8 independent experiments.

We have now performed cTnI promoter analyses in skeletal muscle, a tissue that does not express cTnI [3]. For these experiments we used the regenerating rat soleus muscle, a model that allows efficient gene transfer [5] and can be used for promoter analyses *in vivo *[6-8]. As shown in Figure 1, in regenerating muscle the -230 cTnI/CAT promoter/reporter construct is expressed at very low levels, similar to the levels seen in non muscle cells [4]. The activity of the promoter is neither affected by deletion of the three Sp1 binding elements located between -145 and -127 [4] nor by mutation of the A/T-rich motif. In contrast, mutation of the proximal GATA element (GATA → CACA) surprisingly leads to 5-fold increase in promoter activity and an even greater effect is observed by mutating the more distal tandem GATA elements or all three GATA motifs (13- and 15-fold increase in promoter activity, respectively). To rule out the possibility that this effect is due to generation of new binding sites, we produced two additional mutants: in the first we deleted the two upstream GATA elements in the context of the -230 promoter, in the other we made a different mutation in all three GATA sites (GATA → GCCA). As shown in Fig. 1, promoter activity is also markedly increased in the deleted mutant and in the construct with the GATA → GCCA mutation. The activation of the promoter cannot therefore be due to generation of new binding sites for activating transcription factors, but must result from the lack of a repressive effect of factors binding to these elements.

**Figure 2:**
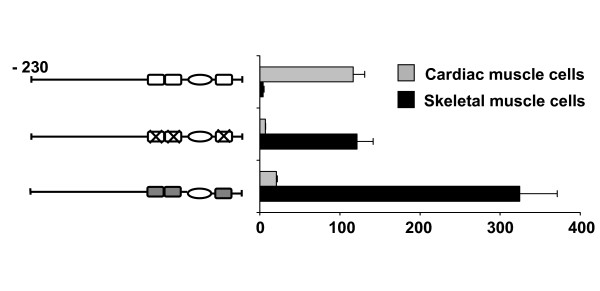
**The cTnI promoter is activated by mutation or deletion of GATA sites in skeletal muscle cells *in vitro***. Transfection experiments of cTnI-CAT constructs in cultured primary cardiomyocytes (grey bars) and skeletal muscle cells (black bars). Schematic drawings of constructs are as in Fig. 1. Note that mutation of GATA sites leads to decreased promoter activity in cardiomyocytes but increased activity in skeletal muscle cells. Values (means ± S.E.) are expressed as fold increase in normalized CAT activity over the promoterless construct. Each construct was tested in 4 to 5 independent experiments.

Next we asked whether the effect of GATA mutations in the cTnI promoter is also observed in cultured skeletal muscle cells. Primary cultures from newborn rat heart and skeletal muscle were transfected with wild-type cTnI promoter and mutated constructs. As shown in Fig. 2, promoter activity was decreased in cardiac but was markedly increased in skeletal muscle cells by mutations of GATA elements. Similar effects were observed with C2C12 cells (not shown).

### cTnI GATA binding activity is present in nuclear extracts of skeletal muscle cells

**Figure 3:**
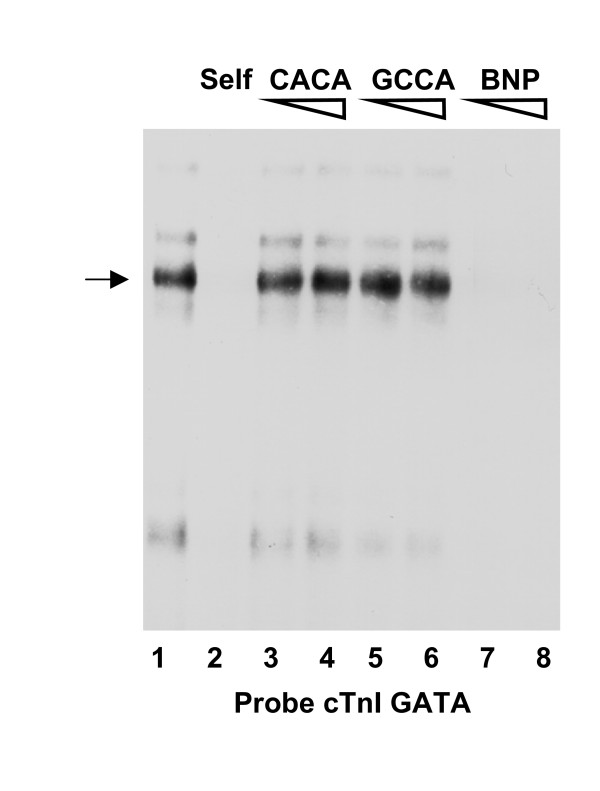
**The cTnI promoter GATA motifs are target for a protein complex in nuclear extracts from C2C12 muscle cells**. A ^32^P-labeled probe encompassing the two distal GATA elements in the cTnI promoter (see Table 1) shows a major retarded complex (arrow) when incubated with nuclear extracts from cultured C2C12 muscle cells (lane 1). For competition assays, the following non-radioactive competitors were added to the reaction mixture at 50- and 100- molar excess prior to the addition of the probe: self competitor (lane 2, 100-fold molar excess), CACA mutated cTnI GATA motifs (lanes 3 and 4), GCCA mutated cTnI GATA motifs (lanes 5 and 6), and a GATA sequence from the BNP promoter (lanes 7 and 8). The formation of the complex is prevented by the cTnI and BNP GATA sequences but is unaffected by the mutated cTnI GATA sequences.

These results suggest that factors binding to GATA elements are present in skeletal muscle cells and are required for repressing the cTnI promoter in these cells. To support this interpretation, we performed electrophoretic mobility shift assays (EMSA) with C2C12 muscle cell nuclear extracts (Fig. 3). Using a DNA probe spanning the two distal GATA sites of the cTnI promoter and nuclear extracts from differentiated C2C12 cells, we identified a major retarded complex (lane 1). The formation of this complex was prevented by self-competition (lane 2) and by GATA sequences from the B-type natriuretic peptide (BNP) promoter (lane 7, 8). In contrast, competition with 50-fold or 100-fold molar excess of double stranded oligonucleotides containing the same mutations in the GATA sites used for promoter analyses (lane 3–6) did not remove the complex. Therefore, a specific GATA binding activity is present in nuclear extracts of C2C12 muscle cells.

### GATA-2, -3 and -4, but not GATA-1, -5 and -6 transcripts are present in skeletal muscle cells

**Figure 4:**
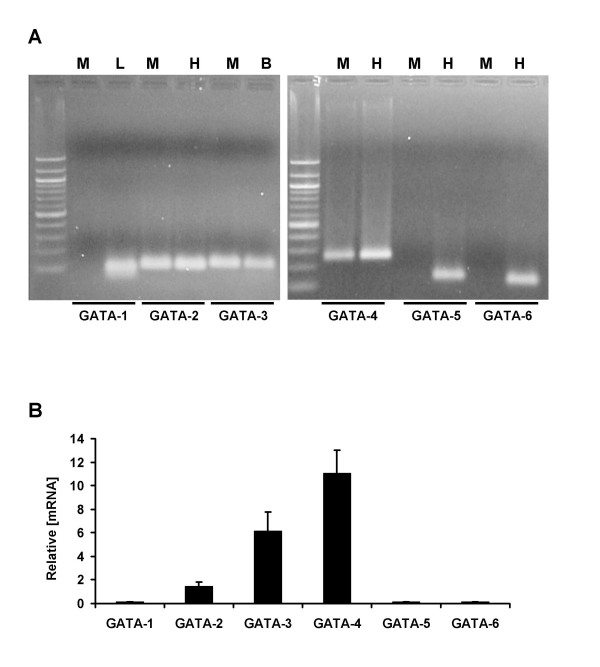
**Muscle cells express GATA-2, -3 and -4 but not GATA-1, -5 and -6 transcripts**. A. RT-PCR assays were performed with RNA preparations from differentiated C2C12 cells (M), using heart (H), liver (L) or brain (B) RNA preparations as positive controls. The primers used are indicated in the Method section. Note that muscle cells express GATA-2, -3 and -4 but not GATA-1, -5 and -6 transcripts. B. Relative mRNA expression levels of GATA transcripts were evaluated by quantitative real-time PCR, using cyclophilin A as an internal standard.

GATA transcription factors have been grouped in two subfamilies based on their expression pattern: GATA-1, -2 and -3 genes are mainly expressed in hematopoietic cells, while GATA-4, -5 and -6 are expressed in various mesoderm- and endoderm-derived tissues, including heart and gut [9-11]. There is no report on the expression of GATA factors in skeletal muscle, except for GATA-2 and -3. GATA-3 transcripts are expressed in the somites at E10, and GATA-3 promoter can also drive lacZ expression in somites at E11.5 [12]. GATA-2 transcript and protein are induced by IGF-1 in skeletal muscle fibers undergoing hypertrophy both *in vitro *and *in vivo *[13-15]. We investigated the expression of GATA factors by RT-PCR in C2C12 muscle cells and detected the presence of GATA-2, -3 and -4 but not GATA-1, -5 and -6 transcripts (Fig. 4A). Quantitative real-time PCR showed that GATA-4 mRNA is relatively more abundant than GATA-3 and GATA-2 in muscle cells (Fig. 4B).

### GATA-2, -3 and -4 proteins are not responsible for cTnI GATA binding activity in skeletal muscle cells

**Figure 5:**
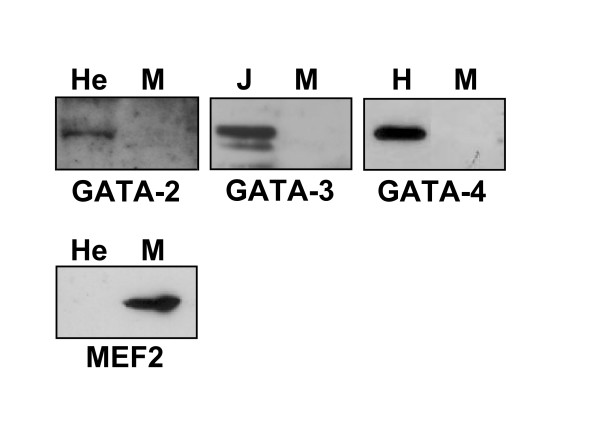
**GATA-3 and -4 proteins are not detected and GATA-2 protein is barely detected by Western blotting in skeletal muscle cells.** Western blotting was performed using antibodies specific for GATA-2, -3 or 4 and nuclear extracts of differentiated C2C12 cells (M) or control cells (He:Hela, J:Jurkat or H:heart cells). A control blot reacted with anti-MEF2 antibody demonstrates the presence of MEF2 in nuclear extracts of muscle cells. 20 μg of extract was loaded per lane.

**Figure 6:**
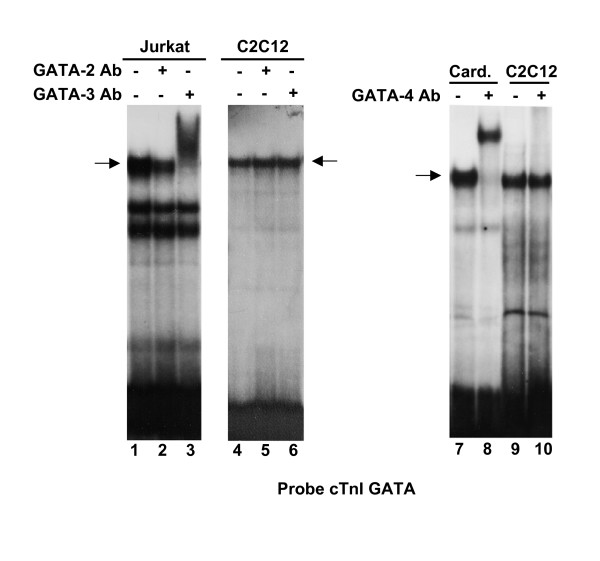
**The cTnI GATA elements do not bind GATA-2, -3 and -4 protein in muscle cell nuclear extracts**. The ^32^P-labeled cTnI GATA probe was incubated with C2C12 nuclear extracts, Jurkat or cardiomyocyte nuclear extracts were used as controls. For supershift experiments, nuclear extracts were preincubated with anti-GATA-2, -3 or -4 antibody. In the presence of the anti-GATA-2 antibody, the major complex observed in the binding reaction of cTnI GATA probe with Jurkat nuclear extracts (arrow) is partially inhibited (compare lanes 1 and 2), while no inhibition is observed with the nuclear extract of C2C12 muscle cells (compare lanes 4 and 5). In the presence of the anti-GATA-3 antibody, a supershift is observed with Jurkat nuclear extract (compare lanes 1 and 3), but no supershift is seen with C2C12 nuclear extract (compare lanes 4 and 6). A supershift is also observed with cardiomyocyte nuclear extract in the presence of the anti-GATA-4 antibody (compare lanes 7 and 8), but no supershift is seen with C2C12 nuclear extracts (compare lanes 9 and 10).

Western blotting analysis was performed to determine whether GATA-2, -3 and -4, which are detected in skeletal muscle cells at the transcript level, are also present at the protein level. However, with the exception of a very faint band observed for GATA-2, these GATA factors were not detected in blots from C2C12 myotubes incubated with specific antibodies (Fig. 5). Gel shift assays were also used to determine whether GATA-2, -3 and -4 proteins are in fact present in skeletal muscle cell nuclear extracts and bind GATA elements of the cTnI promoter. As shown in Fig. 6, the GATA-2 antibody, that partially inhibits the formation of the specific complex in control Jurkat nuclear extract (lane 1, 2), does not significantly change the pattern of gel retardation with C2C12 extract (lane 4, 5). In the presence of the GATA-3 antibody, a clear supershift is observed with Jurkat nuclear extracts (lane 3), whereas the pattern of gel retardation is unchanged with C2C12 extract (lane 6). Finally, a GATA-4 antibody produced a strong supershift with cardiac muscle cell nuclear extract (lane 7, 8) but not with C2C12 cell extract (lane 9, 10).

## Discussion

The results reported here indicate that the cTnI gene promoter is actively repressed in cultured skeletal muscle cells and in regenerating adult skeletal muscle by factors that binds GATA elements. Both mutation and deletion of GATA elements in the cTnI promoter strongly up-regulate promoter activity in skeletal muscle cells. In addition, EMSA experiments show that 1) nuclear extracts of muscle cells contain proteins that form specific complexes with GATA elements present in the cTnI promoter, and 2) these proteins do not correspond to GATA-2, -3 and -4, the only GATA transcripts expressed in skeletal muscle cells. The nature of the GATA-like factor(s) responsible for the repression of the cTnI promoter in skeletal muscle cells thus remains to be established.

A role of GATA elements in the regulation of skeletal muscle gene promoters has been previously proposed. Mutation of a GATA motif in the myosin light chain 3 promoter was found to attenuate its activity in primary cultures of neonatal rat skeletal muscle, however, the nature of the factor(s) binding to this site was not determined [13]. Deletion of a GATA binding sequence in the dihydropyridine-sensitive receptor (DHPR) α1 subunit promoter leads to reduction in promoter activity and antisense experiments for GATA-2 induced functional changes in L-type calcium channels which are consistent with decreased expression of α1 [16]. However, direct interaction of GATA2 with DHPR promoter was not demonstrated. An interaction between GATA factors and MyoD in the regulation of the FGF4 gene promoter in embryonic myotomes has also been described [17]. However, EMSA assays were performed in HeLa cells and no evidence was presented for the presence of GATA factors in the myotomes. Thus direct evidence for the presence of GATA factors in nuclear extracts of skeletal muscle cells was not reported in these studies.

GATA factors are best known as transcriptional activators and inducers of cell differentiation. However, several studies indicate that they can also act as transcriptional repressors in different cell systems, including heart [18], adipose tissue [19] and erythroid cells [20]. Proteins distinct from canonical GATA transcription factors can also bind GATA elements and inhibit gene expression. The ubiquitous nuclear DNA binding protein Ku70 was shown to have a repressor activity in the transcriptional regulation of the GBP and KEL promoters by binding WGATAG, but not WGATAA elements [21,22]. However, it is unlikely that Ku70 is responsible for the repression of the cTnI promoter in skeletal muscle since all of the GATA elements in the cTnI promoter have a GATAA consensus sequence. GATA-like protein-1 (GPL-1), which is expressed at high levels in the somatic cells of the developing gonads, but not in skeletal muscle, acts as a transcriptional repressor of GATA factor function [23].

In conclusion, our results suggest that the transcription of cTnI gene is actively repressed in skeletal muscle cells by proteins binding to GATA motifs in the proximal promoter. The finding that the transcription of the cTnI gene promoter is actively repressed in skeletal muscle cells raises the possibility that under certain conditions this inhibitory mechanisms may be relieved and cTnI transcription may take place also in skeletal muscle. Indeed, cTnI mRNA has been detected in skeletal muscle biopsies of patients with Duchenne muscular dystrophy at the mRNA level [24] though not at the protein level [25].

## Conclusion

The present study shows that cTnI promoter activity is markedly increased in skeletal muscle by mutation or deletion of GATA sites and that these sites form specific complexes with proteins present in nuclear extracts of muscle cells but different from canonical GATA factors. Although the GATA-like factors involved in the repression of the cTnI promoter have not yet been identified, our results suggest a novel mechanism of repression of cardiac promoters in skeletal muscle.

## Methods

### Plasmid constructs

The -230/+16 cTnI promoter linked to chloramphenicol acetyl transferase (CAT) and the mutated constructs have been described [4]. Deletion of the two GATA elements (-67/-57) was obtained by double PCR as described [26]. Briefly, the cTnI promoter was amplified in two separate reactions: from -230 to -67 and from -56 to +16 with the oligos -67 and the -56 designed with a partial overlap. Each PCR product was gel purified and combined in an equimolar ratio. Three cycles without primers were performed, and subsequently 20 cycles of PCR were performed with addition of the 5'-upstream and 3'-downstream primers. The final amplified product was digested with HindIII and SalI and cloned into the plasmid pCAT enhancer (Promega). The construct was confirmed by sequencing.

### Muscle regeneration and in vivo transfection

Male Wistar rats (200–250 g) were anesthetized with Isoflurane (FORANE, Abbott SpA) at 1–1.5% with oxygen. The rat soleus muscle was exposed and muscle degeneration/regeneration was induced by injecting 0.5% bupivacaine (Marcaine) in saline solution. Intramuscular injection of plasmid DNA (50 μg) was performed at day 3 after bupivacaine treatment as described previously [5]. Muscles were removed at 7 days after injury and frozen in isopentane cooled in liquid nitrogen. Animals were kept in conventional facilities with free access to food and water. Adequate care for their health and well-being was provided in accordance with the Italian Animal Act (Law 116/92). These studies were conducted under the supervision of the Institutional Ethics Committee.

### Muscle cell cultures and transfections

Primary cultures of cardiac and skeletal muscle cells were prepared from neonatal rat heart and hind limb muscles as described previously [27]. Skeletal muscle cells were grown for 3 days in DMEM supplemented with 20% fetal calf serum, 2% glutamine and 12.5 U/ml penicillin-streptomycin, then transfected with 10 μg of the cTnI-CAT constructs and 1 μg of CMV-lacZ, as an internal control. After 24 h, the cells were induced to differentiate by changing the medium to DMEM supplemented with 4% horse serum, 2% glutamine, 12.5 U/ml of penicillin-streptomycin. The cultures were harvested after 2 days in the differentiation medium. A similar procedure was used to obtain differentiated cultured from C2C12 cells.

### CAT assays

Muscles were homogenized in 200 μl ice-cold lysis solution containing 100 mM potassium phosphate pH 7.8, 0.1% triton and 1 mM dithiothreitol (added just before use). Tissues were homogenized with an Ultra Turax T25. The homogenates were centrifuged at 10.000 rpm for 30 min at 4°C; the supernatants were frozen in liquid nitrogen and stored at -80°C. CAT activity was assayed by TLC scintillation counts as described [4]. Values (means ± S.E.) were expressed as fold increase in CAT activity over the promoterless construct. Each construct was tested in 4 to 8 independent experiments. For muscle cell cultures β-Galactosidase and chloramphenicol acetyl transferase (CAT) assays were performed with 25 μl of muscle cell lysate. Values (means ± S.E.) were expressed as fold increase in normalized CAT activity over the promoterless construct. Each construct was tested in 3 to 5 independent experiments.

### Real-time RT-PCR

For real-time PCR assays, total RNA was prepared from C2C12 myotubes with SV Total RNA Isolation reagent (Promega). 400 ng of RNA was converted to cDNA using random hexamers and Superscript II (Invitrogen). PCR amplification was performed with an IQ5 real time PCR system (BioRad) using SYBR green. The primers were designed to span an intronic sequence and were validated by PCR and gel analysis. Primer sequences were as follows (forward & reverse):

GATA-1

F: 5' GCTCAGCAGCCTATTCTTCC 3'

R: 5' CGTTGCTCCACAGTTCACAC 3'

GATA-2

F: 5' ACGCCTGTGGCCTCTACTAC 3'

R: 5' GGATTTGCTGGACATCTTCC 3'

GATA-3

F: 5' ACCACGTCCCGTCCTACTAC 3'

R: 5' AGAGATCCGTGCAGCAGAG 3'

GATA-4

F: 5' CTGTCATCTCACTATGGGCAC 3'

R: 5' CCAAGTCCGAGCAGGAATTTG 3'

GATA-5

F: 5' TTTGAAGGCAGAGTCCAGTC 3'

R: 5' AGGCAAAGTCTTCAGGTTCG 3'

GATA-6

F: 5' TTCTACACAAGCGACCACCTC 3'

R: 5' GCCGTCTTGACCTGAATACTT 3'

The data were normalized using oligonucleotide primers for cyclophilin A as an internal standard.

### Western blotting

Nuclear extracts (20 μg) from C2C12 myotubes or other cell types were separated by SDS-PAGE using 8% gels and blots were incubated with monoclonal antibodies specific for GATA-2, -3, -4 and a polyclonal antibody specific for MEF2 (Santa Cruz Biotechnology Inc.). Antibody binding was revealed by use of SuperSignal West Pico chemiluminescent substrate (Pierce).

### Gel Mobility Shift Assay

DNA/protein reactions and electrophoretic mobility shift assays (EMSA) were performed as described [28] using 0.3 ng of cTnI GATA radiolabeled probe, 1 μg of poly (dI·dC)-poly(dI·dC) (Amersham-Pharmacia Biotech) and a 50–100-fold excess of competitor DNA. We used 2 μg of nuclear extracts from C2C12 muscle cells and either Jurkat cells (Geneka) or cultured cardiomyocytes. Supershift assays were performed with antibodies specific for GATA-2, -3 or -4 (Santa Cruz Biotechnology Inc.). The antibodies were added to the DNA/nuclear extract mix, and the binding was allowed to proceed for 1 hour before loading the samples. The oligonucleotides used for these experiments, including one specific for GATA sequences from the BNP promoter [29], are listed in Table 1.

**Table 1:** Oligonucleotides used in gel shift assays *

cTnI GATA^#^	5' CGCCTGTTATCGCTTATCCTGGG 3' GCGGACAATAGCGAATAGGACCC
cTnI mut GATA → CACA	5' CGCCTGTT**G**T**G**GCTT**G**T**G**CTGGG 3' GCGGACAA**C**A**C**CGAA**C**A**C**GACCC
cTnI mut GATA → GCCA	5' CGCCTGTT**GG**CGCTT**GG**CCTGGG 3' GCGGACAA**CC**GCGAA**CC**GGACCC
BNP GATA	5'GATCCCAGGAATGTGTCTGATAAATCAGAGATAACCCA 3'

* GATA sites are underlined. Mutated nucleotides are indicated in bold.

^# ^This sequence comprises the two GATA elements closely associated between -67 and -57 of the cTnI promoter

### Data analysis

Data are expressed as means ± SE (represented as error bars). Comparisons were made using the Student's *t *test, considering P < 0.05 statistically significant.

## Authors' contributions

RDL performed most of the experiments and contributed to the analysis and interpretation of the data. AP performed RT-PCR and some of the gel shift, CAT assays and Western blotting. SA contributed to the design of the experiment. SS planned and supervised the experiments and wrote the paper with input from RDL and SA. All authors read and approved the final manuscript. 
